# Excluded GM-specific IgG Subclass Genes in Health and Disease - Inborn Errors of Immunity

**DOI:** 10.1007/s10875-026-02001-5

**Published:** 2026-03-21

**Authors:** Vivi-Anne Oxelius

**Affiliations:** 1 Department of Pediatrics, Institute of Laboratory Medicine, University Hospital, Lund University, Lund, Se-221 85 Sweden; 2 Department of Clinical Immunology, University Hospital, Lund University, Lund, Sweden

**Keywords:** Precise alternative GM-specific Fc parts of heavy constant G chains of IgG3, IgG1 and IgG2 subclasses, Respectively: IgG3*b & IgG3*g, IgG1*f & IgG1*a and IgG2*n & IgG2*-n on chromosome 14q32.3, By mendelian inheritance the homozygous genotypes have excluded alternative IgG subclass genes with total loss of alternative IgG subclass molecules, Inborn errors of immunity, Significantly increased in immunological disease, With IVIG as possible treatment

## Abstract

**Purpose:**

IgG subclass genes from chromosome 14q32.3 are assessed serologically by GM allotypes, genetic markers of the Fc part of the immunoglobulin constant heavy G chains, (*IGHG*(Fcγ)(GM) genes. Alternative GM allotypes of IgG3, IgG1 and IgG2, respectively, define 6 unique, precise GM-specific IgG subclass genes, inherited the Mendelian way with allelic exclusion, and linkage disequilibrium of IgG3-IgG1. Increased number of homozygous GM-specific IgG subclass genotypes with total loss of alternative IgG subclass molecules, inborn errors of immunity (IEI), are found in severe immunological diseases.

**Methods:**

A novel ELISA using GM-specific myeloma proteins and GM-specific monoclonals, identifies 6 alternative GM-specific IgG subclass genes and molecules IgG3*b & IgG3*g, IgG1*f & IgG1*a and IgG2*n & IgG2*-n, with different structures and functions. 4 different IgG3-IgG1-IgG2 haplotypes encode 10 individual *IGHG* diplotypes from 10 innate lymphoid combined B cells in 587 healthy.

**Results:**

The alternative GM-specific IgG subclass genes have different structures and functions and respond differently in immunotherapy to antigen stimulation with virus, bacteria and allergens. In this report we focus on excluded GM-IgG subclass genes, inborn errors of immunity (IEI) dominating in severe immunological diseases, severe infections, primary immunodeficiencies (PIDs), JCA, asthma, diabetes type 1 and malignancy. By intravenous immunoglobulins (IVIG) the excluded IgG subclass molecules are supplied and may prevent primary virus attacks and exacerbations in autoimmune disorders. IgG subclass genes orchestrate additional immune factors of inflammation.

**Conclusion:**

Excluded GM-specific IgG subclass genes, IEI explore diagnosis, pathogenesis, prognosis and different phenotypes in immunological diseases, with IVIG as treatment.

## Introduction

**Fig. 1:**
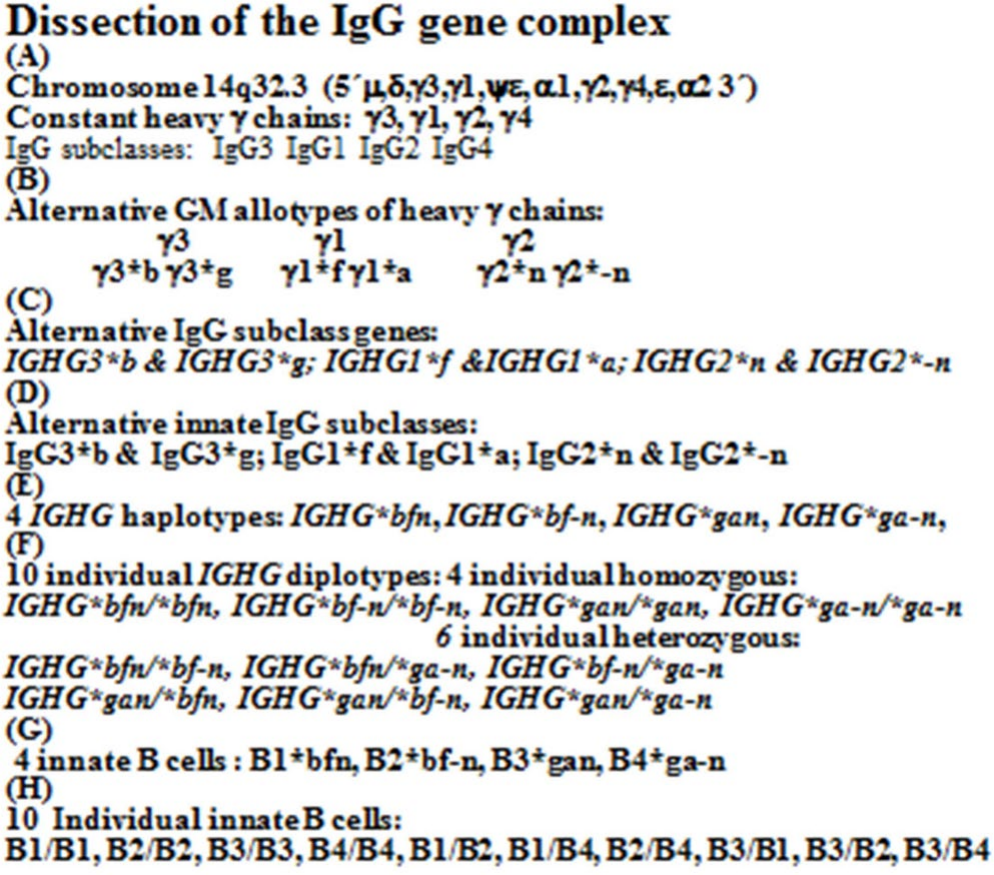
Serologic dissection of *IGHG*(Fcý)(GM) genes, innate IgG subclasses and innate B cells

GM allotypes are genetic markers of the Fc part of constant heavy G chains [1, 2] encoding IgG subclass genes, *IGHG*(Fcγ)(GM) genes from chromosome 14q32.3 ( 5´µ, δ, γ3, γ1,ψε, α1, γ2, γ4, ε, α2 3´) (Fig. 1). IgG subclass genes are investigated serologically by GM allotypes [3, 4]. The IgG subclasses IgG3, IgG1 and IgG2 (not IgG4) are differentiated by alternative GM allotypes: IgG3*b & IgG3*g, IgG1*f & IgG1*a and IgG2*n & IgG2*-n.

The alternative GM specific IgG subclass molecules are unique precise molecules and have structural and functional differences [5]. The alternative IgG subclass genes, are found with amino acid substitutions in IGHG1*a/IGHG1*f at CH1(214) Lys/Arg, at CH3(356) Asp/Glu, at CH3(431)Gly/Ala and in IGHG2*n/IGHG2*-n at CH2(282) Met/Val, and immunochemical differences: different electrophoretic rates [5], different half-life-times [6], different age-dependent developing rates [8], and different antibody responses by antigen stimulation [9] virus [10], bacteria [11] and allergens [12].

**Fig. 2:**
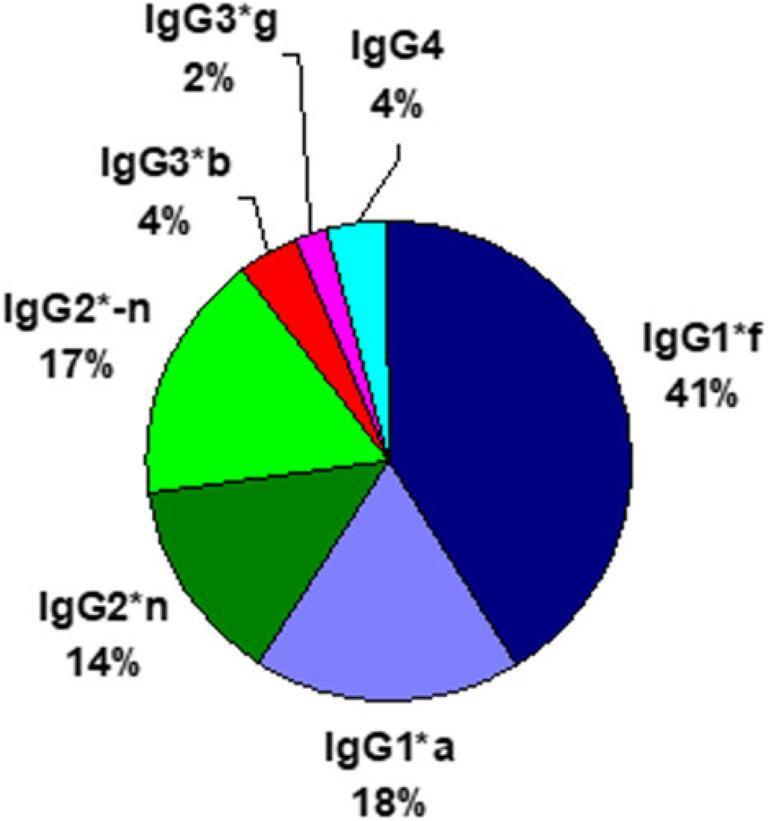
Proteomics. Amounts (%) of 6 innate IgG subclasses in a normal serum pool (c: a 2000 sera): IgG1*f (41%), IgG1*a (18%); IgG2*n (14%), IgG2*-n (17%) and IgG3*b (4%) & IgG3*g (2%). No genetic variation in IgG4

**Fig. 3:**
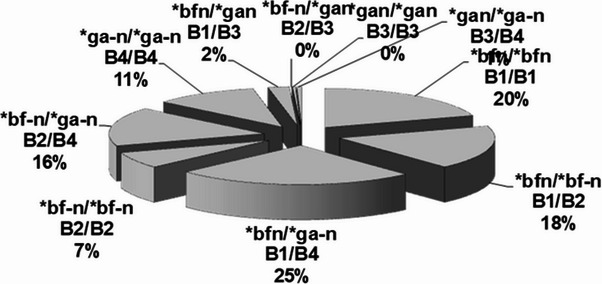
Gene map of a healthy Caucasian population (587). 10 Individual diplotypes of *IGHG*(Fcý)(GM) genes and 10 combined innate B cells (%)

**Fig. 4:**
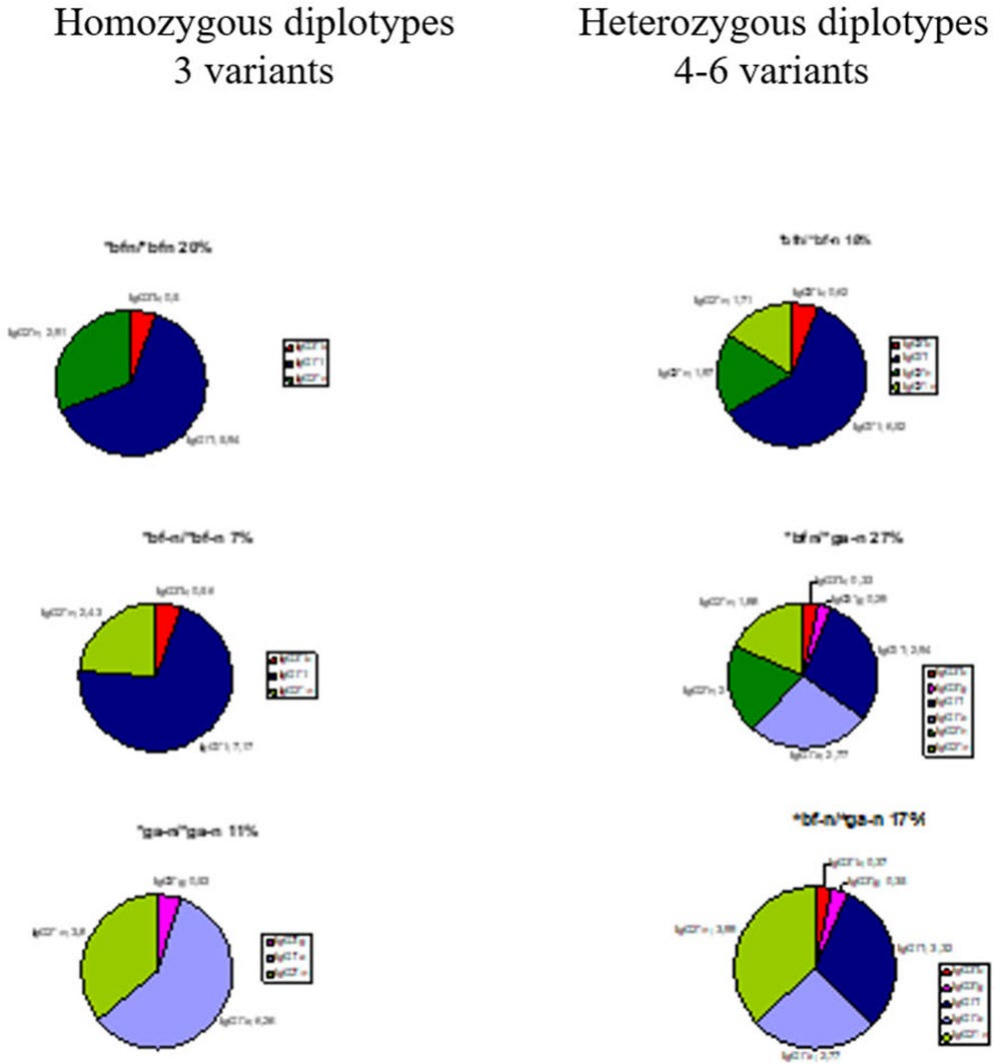
Genomics & Proteomics of IgG subclass diplotypes in healthy individuals. Qualities of IgG subclass genes and quantities innate IgG subclass molecules. Homozygous (3 variants) and heterozygous (4–6 variants) diplotypes in 157 adult Caucasians

GM-specific IgG subclass genes are inherited according to Mendel with allelic exclusion and γ3-γ1 linkage disequilibrium. In homozygous genotypes, the alternative GM specific IgG subclass genes are excluded, with total loss of alternative GM-specific IgG subclass molecules, IEI (Table 1). 4 different GM specific IgG3-IgG1-IgG2 haplotypes encode 10 individual diplotypes and are genetic markers of 10 innate lymphoid B cells [7] (Fig. 1; Table 1). Of the 10 diplotypes 4 are homozygous with only 3 GM-specific IgG subclass gene variants and 6 are heterozygous with 4–6 GM-specific IgG subclass genes (Figs. 2, 3 and 4).

**Table 1:** GENE MAP. Individual sera with expressed *IGHG*(Fcý)(GM) diplotypes, innate B cells and innate IgG subclasses in 587 healthy Caucasians (number, %). Excluded alternative GM specific IgG subclass genes in homozygous genotypes with total loss of innate IgG subclasses

Individual IGHG(Fcý) (GM) diplotypes In 587 healthy	*bfn/ *bfn	*bfn/ *bf-*n*	*bfn/ *ga-*n*	*bf-*n*/ *bf-*n*	*bf-*n*/ *ga-*n*	*ga-*n*/ *ga-*n*	*bfn/ *gan	*bf-*n*/ *gan	*gan/ *gan	*gan/ *ga-*n*
B cells	B1/B1	B1/B2	B1/B4	B2/B2	B2/B4	B4/B4	B1/B3	B2/B3	B3/B3	B3/B4
Innate IgG subclasses	IgG3*b IgG1*f IgG2*n	IgG3*b IgG1*f IgG2*n IgG2-n	IgG3*b IgG3*g IgG1*f IgG1*a IgG2*n IgG2*-n	IgG3*b IgG1*f IgG2*-n	IgG3*b IgG3*g IgG1*f IgG1*a IgG2*n	IgG3*g IgG1*a IgG2*-n	IgG3*b IgG3*g IgG1*f IgG1*a IgG2*n	IgG3*b IgG3*g IgG1*f IgG1*a IgG2*n IgG2*-n	IgG3*g IgG1*a IgG2*n	IgG3*g IgG1*a IgG2*n IgG2*-n
Number	118	107	147	40	95	84	13	0	2	1
**%**	20.1%	18.2%	25.0%	6.8%	16.2%	10.9%	2.2%	< 1%	0.3%	0.2%
Excluded *IGHG* subclass genes in homozygous genotypes	IGHG3*g IGHG1*a IGHG2*-n	IGHG3*g IGHG1*a	None	IGHG3*g IGHG1*a IGHG2*n	IGHG2*n	IGHG3*b IGHG1*f IGHG2*n	IGHG2*-n	None	IGHG3*b IGHG1*f IGHG2*-n	IGHG3*b IGHG1*f
Total loss of innate IgG subclasses	IgG3*g IgG1*a IgG2*-n	IgG3*g IgG1*a	None	IgG3*g IgG1*a IgG2*n	IgG2*n	IgG3*b IgG1*f IgG2*n	IgG2*-n	None	IgG3*b IgG1*f IgG2*-n	IgG3*b IgG1*f

*IGHG*(Fc)(GM) genes are associated with immunological diseases [9], significantly increased in severe infections both viral and bacterial, primary immunodeficiencies (PID)s [13, 14], allergy [12, 15], severe autoimmunity [16, 17] and malignancy [18, 19].

A novel serological competitive enzyme-linked assay (ELISA) technique is favorable, utilizing GM specific reagents, purified GM specific myeloma proteins and GM specific monoclonals [3, 4] assessing the complex inheritance of individual *IGHG*(Fcγ)(GM) genes qualitatively and quantitatively. 6 genes (genomics) (Fig. 2) and 6 precise unique innate IgG subclass molecules (proteomics) are identified, quantitatively (Fig. 3). The serum pool (2000 sera) contains 6 innate GM-specific IgG subclasses (%) IgG1*f (41%), IgG1*a (18%), IgG2*n (14%), IgG2*-n (17%), IgG3*b (4%) and IgG3*g (2%) and IgG4 (4%) (Fig. 3).

In homozygous IgG subclass genotypes the alternative IgG subclass genes are excluded with total loss of alternative IgG subclass molecules (Table 1), inborn errors of immunity (IEI). The individual *IGHG*(Fcγ)(GM) genes with origin from parental GM markers, of chromosome 14q32.3 are present in all somatic cells [21], expressed by B cells. 10 individual diplotypes, 4 homozygous and 6 heterozygous *IGHG*(Fcγ)(GM) diplotypes together with innate lymphoid B cells are typed in the 587 healthy Caucasians (Fig. 3). *IGHG*(Fcγ)(GM) genes have impact on severe immunological diseases, different phenotypes of diseases, and on active and passive immunotherapy. The qualities and quantities of *IGHG*(Fcγ)(GM) genes are given in 587 healthy Cauucasians (Table 1). The novel ELISA serological methods have advantage by mapping *IGHG*(Fcγ) (GM) diplotypes, innate lymphoid B cells and innate IgG subclasses qualitatively quantitatively, subsequently [3, 9]. The Mendelian *IGHG* genes are found in all human somatic cells, also stem cells [21]. They respond differently in vaccination high responding or low responding [13]. There is a risk of allo-immunization in passive immunotherapy by humanized recombinant monoclonal antibodies, using restrictive variants of allelic IgG1antibodies for patients with various *IGHG*(Fcγ)(GM) diplotypes. The variable GM-specific IgG subclass genes with different phenotypes have impact in pandemics. The excluded *IGHG*(Fcγ)(GM) genes in Mendelian inheritance with total loss of IgG subclass molecules, are also found in healthy was inborn errors of immunity, risk genes. In this report we focus on excluded GM-specific IgG subclass genes with total loss of GM-specific IgG subclass molecules, inborn errors of immunity (IEI), in health and disease.

## Materials

Serum samples of 587 healthy (Fig. 1) (Table 1) both children and adults [3] were investigated. Sera from an additional healthy group of 50 mother-father-child confirmed the inheritance pattern. >383 sera from patients with different diseases were investigated: 49 sera from children with RS virus infection, 49 sera from children with Kawasaki syndrome 233 patients with PIDs, 76 children with JCA, 36 children with diabetes type 1 and different groups of IgE mediated allergy and infection asthma.

## Methods

A novel competitive enzyme-linked immunosorbent ELISA assay [3, 8, 9] with the following monoclonal antibodies: anti IgG1*f clone 5F10, anti IgG1*a clone 5E7, anti IgG3*b1/u clone 12D9 (Janssen Biochimica, Beerse Belgium) and anti IgG2*n clone SH21 (Sigma, St.Louis. MO, USA). Microtitre plates were coated with a predetermined allotypes: concentration of purified myeloma protein of the following IgG allotypes: IgG1*f, IgG1*a, IgG2*n and IgG3*b. A normal diluted serum pool (about 2000 sera) was used and a panel of GM specific purified myeloma proteins was utilized as positive and negative controls. Determination of homozygosity and heterozygosity for the IgG2*n allotype was done with a double immunodiffusion assay with the monoclonal reagents anti IgG2*n SH21 and anti HP clone USA6014 (Sigma, St Louis MO, USA) [3, 4]. The sensitivity of the ELISA for serum innate IgG subclasses: 0.0003 g/l for IgG1*f, 0.0008 g/l for IgG1*a, 0.0006 g/l for IgG2*n, 0.0007 g/l for IgG3*b.

The control test for IgG3*g, beside absence of IgG3*b, was also the concordant IgG1*a allotype. The quantities of GM specific innate IgG subclasses were calculated from knowledge of the gene frequencies in a large Caucasian population of more than 2000 serum samples, with given proportions of the GM allotypes within the IgG subclasses for IgG3*b/IgG3*g, 0.51/0.22 g/l, 70/30, for IgG1*f/IgG1*a 4.76/2.04 g/l 70/30 and for IgG2*n/IgG2*-n 1.5/1.83 g/l 45/55 [3, 5] (Fig. 2). By the ELISA tests the quantitative expressions of *IGHG* genes are available. Total IgG subclass levels were confirmed with the Mancini technique. The GM specific IgG subclass gene levels of the *IGHG* diplotypes of 157 healthy adults and the developmental rate during childhood has been given (mean +/- SD) as references [8]. The method to study genes by serum protein copies is advantageous as the protein quantities in serum also disclose the rate of gene activity. The ELISA method is the simplest way to demonstrate GM specific IgG subclass genes, by quality and quantity, in health and disease. DNA studies as genome wide association studies (GWAS) and Hap Map have not involved GM allotypes in their genotyping platforms and did not detect the *IGHG*(Fcγ)(GM) genes as candidate genes.

## Results

### Excluded IgG Subclass Genes in Healthy- Gene Map

**Fig. 5:**
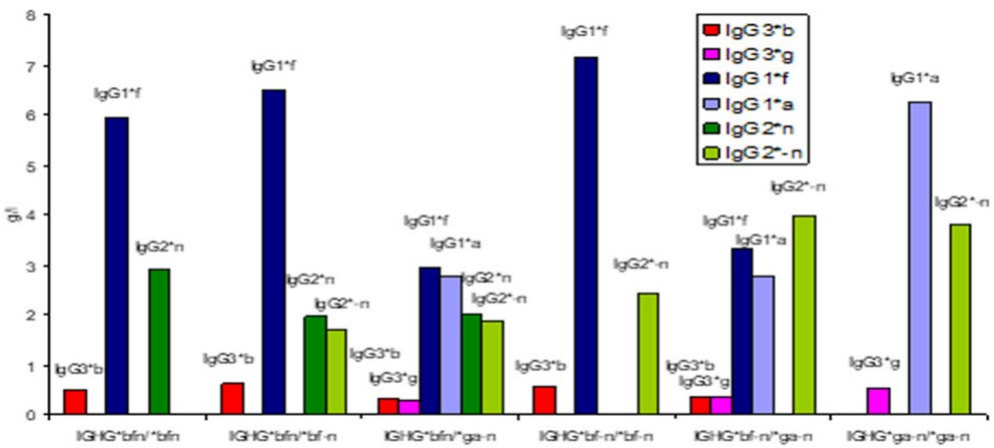
Serum levels of innate IgG subclasses (mean level, g/l) in the 6 most common *IGHG*(Fcý)(GM) diplotypes of 157 adult healthy Caucasians. Homozygous genotypes with 3 innate IgG subclasses and heterozygous genotypes with 4–6 innate IgG subclasses

GM specific IgG subclass genes are associated with severe immunological diseases, published earlier [9]. In this report we focus on excluded IgG subclass genes. In healthy two alternative GM allotypes encode IgG3, IgG1 and IgG2, respectively (Fig. 1) on chromosome 14q32.3, 6 IgG subclass genes: IGHG3*b & IGHG3*g, IGHG1*f & IGHG1*a and IGHG2*n & IGHG2*-n, genomics, identified serologically by GM alleles on unique precise IgG subclasses: IgG3*b & IgG3*g, IgG1*f & subclassIgG1*a and IgG2*n & IgG2*-n, proteomics (Fig. 1). GM-specific IgG subclass genes are inherited the Mendelian way, with allelic exclusion and IgG3-IgG1 linkage disequilibrium. Of the 10 individual diplotypes in the healthy Caucasian population (587) 4 diplotypes are homozygous: Homozygous IGHG*bfn/*bfn, with excluded alternative IGHG3*g, IGHG1*a and IGHG2*-n genes, homozygous IGHG*bf-n/*bf-n, with excluded alternative IGHG3*g, IGHG1*a and IGHG2*n genes, homozygous IGHG*gan/*gan (rare) and homozygous IGHG*ga-n/*ga-n with excluded alternative IGHG3*b, IGHG1*f and IGHG2*n genes (Fig. 4; Table 1 ). The 6 heterozygous diplotypes contain a few homozygous genotypes: IGHG*bfn/*bf-n, with homozygreous IGHG*bf/*bf genotypes revealed excluded IGHG3*g and IGHG1*a genes and IGHG*bf-n/*ga-n with homozygous IGHG2*-n/*-n revealed excluded subclass IGHG2*n genes (Fig. 4). Homozygous GM-specific IgG subclass genes with excluded alternative GM- specific IgG subclass genes and total loss of GM-specific IgG subclass molecules are IEI with increased susceptibility of severe infections and severe immunological diseases (Fig. 5).

**Fig. 6:**
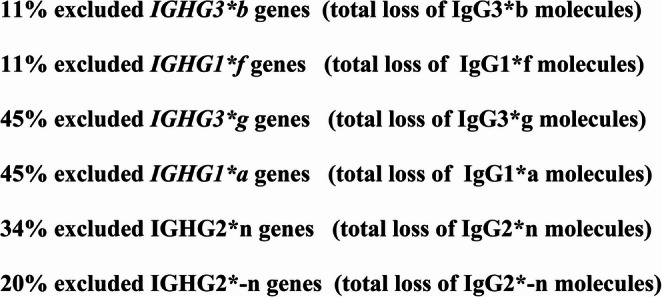
% Excluded IgG subclass genes with total loss of innate IgG subclasses found in a healthy Caucasian population (587), inborn errors of immunity

Excluded *IGHG*(Fcγ)(GM) genes with total loss of IgG subclass molecules are found in 587 healthy Caucasians: 10.9% loss of IgG3*b & IgG1*f, 45.1% loss of IgG3*g & IgG1*a, 33.9% loss of IgG2*n & 20.1% loss of IgG2*-n molecules (Fig. 6). Different excluded IgG subclass genes with total loss of innate IgG subclasses dominate in severe viral and bacterial infections PIDs autoimmune diseases allergy and malignancy. Excluded IgG subclass genes and total loss of innate IgG subclasses is IEI in healthy individuals with increased risk to fall ill. The variable *IGHG*(Fcý)(GM) genes (Table 1) have impact on different phenotypes in pandemics.

## Excluded *IGHG* Genes in Diseases

### Excluded IGHG2*n Genes

#### In RS Virus Infection

49 children with severe respiratory syncytial virus, (RSV), are found with significantly increased frequency, 55%, homozygous IGHG2*-n/*-n genotypes with excluded IGHG2*n genes and total loss of innate IgG2*n molecules [10]. The IGHG2*-n gene is found in a dose dependent way (Table 2). The IGHG2*-n genes are risk genes, while IGHG2*n genes are protective. *IGHG2* genes renders different severity in viral diseases with homozygous IGHG2*-n/*-n, excluded IGHG2*n and loss of IgG2*n antibodies, as the most severe. Symptom variation are recognized in pandemics (Table 3 and 4).

**Table 2:** Excluded IGHG2*n genes and loss of IgG2*n antibodies in RSV infection and Kawasaki syndrome

RSV infection and Kawasaki syndrome have significantly increased frequencies of homozygous IGHG2*-*n*/*-*n* genotypes with excluded IGHG2*n genes and loss of IgG2*n antibodies				
IGHG2 genes	Homozygous IGHG2 *-*n*/*-*n*	Heterozygous IGHG2 *-*n*/*n	Homozygous IGHG2 *n/*n	Excluded IGHG2*n genes and total loss of IgG2*n antibodies
RSV(49)	55%	34.6%	10.2%	55%
Kawasaki(49)	**55%**	**35%**	**10%**	**55%**
Healthy (587)	**34%**	**44%**	**22%**	**34%**

**Table 3:** Exclued IGHG2*n genes and loss of IgG2*n antibodies in PIDs

235 children and adult patients with various primary immundeficiencies (PIDs) have significantly increased frequencies of homozygous IGHG2*-*n*/*-*n* genotypes with excluded alternative IGHG2*n genes and loss of IgG2*n antibodies [14]				
IGHG2 genes	Homozygous IGHG2 *-*n*/*-*n*	Heterozygous IGHG2 *-*n*/*n	Homozygous IGHG2 *n/*n	Excluded IGHG2*n genes and total loss of IgG2*n antibodies
CVID(33)	76%	15%	9%	76%
IgG2D(43)	**58%**	**12%**	**30%**	**58%**
IgG3D(54)	**57%**	**33%**	**9%**	**57%**
IgAD(62)	**55%**	**34%**	**11%**	**55%**
WAS(21)	**67%**	**29%**	**5%**	**67%**
A-T(22)	**45%**	**50%**	**5%**	**45%**
Healthy(587)	**34%**	**44%**	**22%**	**34%**

**Table 4:** Excluded IGHG2*a genes and loss of IgG2*a antibodies in diabetes type 1

36 children with diabetes type 1 have significantly increased frequencies of homozygous IGHG1*f/*f genotypes with excluded IGHG1*a genes and loss of IgG1*a antibodies.				
IGHG1 genes	Homozygous IGHG1 *f/*f	Heterozygous IGHG1 *f/*a	Homozygous IGHG1 *a/*a	Excluded IGHG1*a genes and total loss of IgG1*a antibodies
diabetes type 1	59%	37%	6%	59%
Healthy (587)	**45%**	**43%**	**11%**	**45%**

#### In Kawasaki Syndrome

49 patients with Kawasaki syndrome, (Table 2) picked from a Swedish study [26] are dependent on the IGHG2*-n genes, with the highest frequency in homozygous IGHG2*-n/*-n genotypes (55%), heterozygous IGHG2*-n/*n (35%) and alternative homozygous IGHG2*n/*n (10%). Kawasaki syndrome has been found as a severe phenotype of Covid-19 [27]. Our Kawasaki patients are found with significantly increase of homozygous IGHG2*-n/*-n with excluded IGHG2*n genes and loss of IgG2*n molecules (Table 2) to be compared with the gene pattern of severe RS viral disease. Early IVIG treatment is sucessful in Kawasaki syndrome, with supply of the lost IgG2*n molecules, shown in PID patients (Fig. 6).

### In Primary Immunodeficiencies (PID)s

**Fig. 7:**
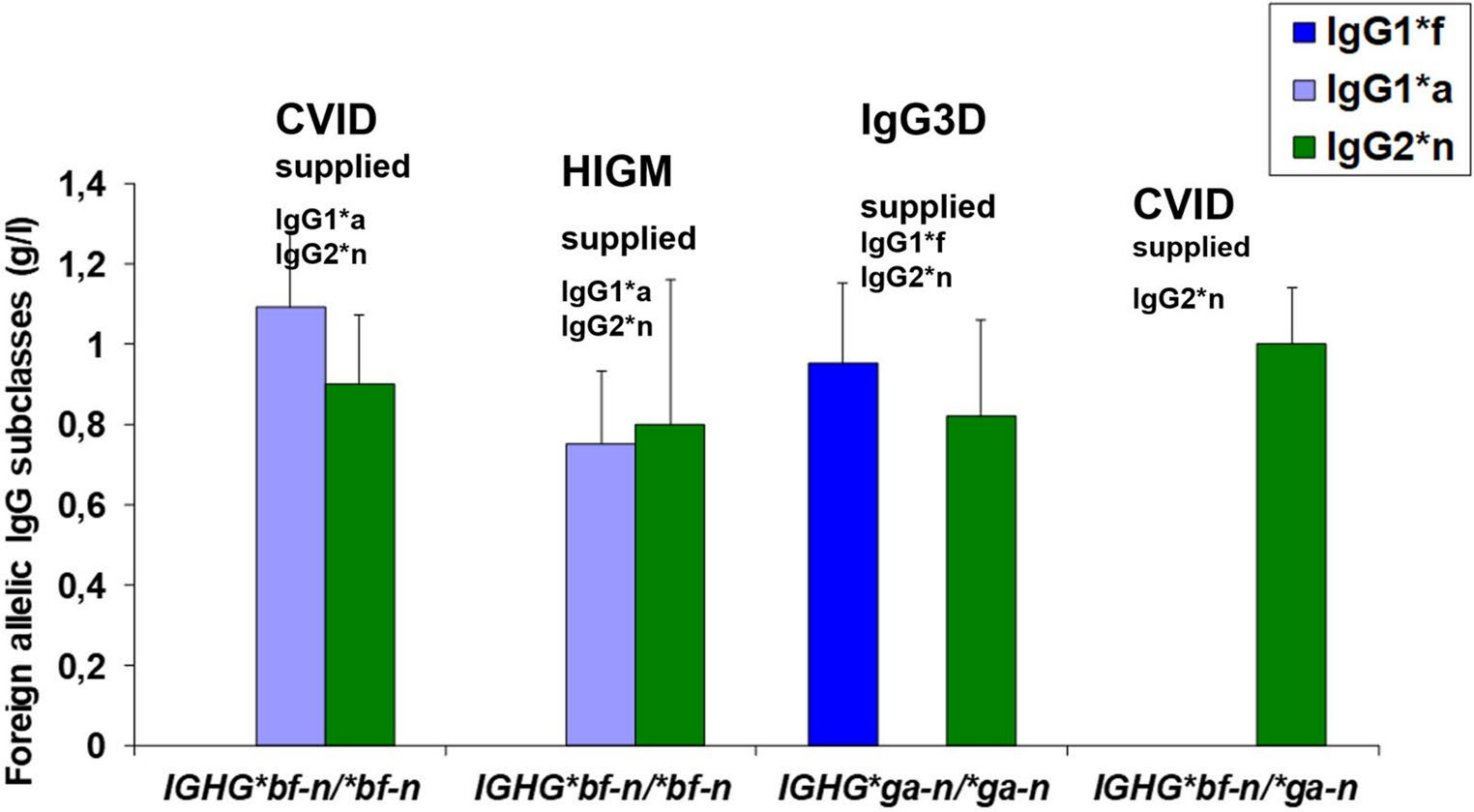
Supply of excluded IgG subclass genes (GM-sgenespecific IgG subclass molecules) by IVIG ireatment of 4 patients with PIDs and excluded GM-specific IgG subclass genes. Infusion with IVIG given intravenously every 4th week at 400 mg/kg body weight. Mean of trough levels, 8 observations

Excluded IGHG2*n genes should not be mixed up “IgG2 subclass deficiency [13], which is defined as significantly low serum levels of the serum IgG2 subclass. The novel ELISA method investigating *IGHG*(Fcý)(GM) genetic variants is preferable, with the possibility to assess excluded IGHG2*n genes and total loss of innate IgG2*n GM-specific IgG subclasses. IgG subclass deficiencies are found to have excluded genes, and loss of innate IgG antibodies [13]. IgG2 deficiency (IgG2D) has increased frequency of homozygous IGHG*bf-n/*bf-n diplotypes, excluded IGHG3*g, IGHG1*a and IGHG2*n genes and loss of innate IgG3*g, IgG1*a and IgG2*n antibodies. IgG3 deficiency (IgG3D) has increased frequency of homozygous IGHG*ga-n/*ga-n diplotypes, excluded IGHG3*b-, IGHG1*f- and IGHG2*n genes and loss of innate IgG3*b, IgG1*f and IgG2*n antibodies, both with significant loss of innate IgG*n antibodies. Significantly increased frequency of the IGHG2*-n/*-n genotypes, excluded IGHG2*n genes and loss of innate IgG2*n antibodies are found in variable immunodeficienctes (CVID) (76%), IgA deficiency (IgAD) (55%) together with the monogenic characteristics found in Wiscott-Aldrich syndrome (67%) and Ataxia Telangiectasia [14] (Fig. 9B). Of 233 PIDs 129, 59% are found with homozygous IGHG2*-n/*-n genotype and excluded IGHG2*n genes and total loss of innate IgG2*n molecules. Loss of IgG2*n genes are probably the most important component for infection susceptibility both viral and bacterial. Excluded *IGHG*(Fcý)(GM) genes in PIDs are supplied with missing IgG molecules by IVIG treatment (Fig. 7).

#### In Severe Encapsulated Bacterial Infections

The homozygous IGHG2*-n/*-n genotypes are low specific antibody responding and with increased susceptibility to encapsulated bacterias compared to the opposite IGHG2*n/*n, high responding [11, 13]. 34% of healthy have excluded IGHG2*n genes with risk to fall ill by severe encapsulated bacteria also worsening viral disease [3]. Excluded IGHG2*n genes may be one explanation of why healthy individuals suddenly fall ill in septicemia. IVIG containing all Fcý gene variants. Supply of missing IgG antibodies is a proposed treatment.

#### In Juvenile Chronic Arthritis (JCA), the Systemic Form

IgG is thought to play a central role in severe systemic juvenile chronic arthritis (JCA). The serum hyper IgG of 76 JCA patients demonstrated mainly increased innate IgG1*a levels from the dominating IGHG*ga-n/*ga-n genotypes in the most severe form, the systemic form of JCA [17]. The alternative genotype IGHG*bfn/*bfn diplotype is uncommon but have a higher rate of remission and a more favorable outcome. In the systemic form JCA, the frequency of the homozygous IGHG2*-n/*-n genotypes were increased with excluded IGHG2*n genes and loss of innate IgG2*n antibodies [17], to be compared to the primary immunodeficiencies.

#### In Malignancy

Homozygosity of IGHG*ga-n/*ga-n genotypes with excluded IGHG2*n genes are found dominating in non-small cell lung cancer [28].

#### In non IgE Mediated Infection Asthma

Children with bronchial asthma with IgE < 10 kU/l, are associated with the homozygous IGHG*ga-n/*ga-n diplotype, infection proness and excluded IGHG2*n molecules. IgG subclass genes are a way of diagnosing various forms of asthma in children. The *IGHG*(Fcý)(GM) genes of chronic lung disease in adults is not known.

### Excluded IGHG2*-n Genes

#### In IgE Mediated Bronchial Asthma

Severe IgE mediated allergy and asthma in children are associated with the homozygous IGHG*bfn/*bfn diplotype and total loss of IgG2*-n. IgE sensitization is dependent on the IGHG2*n gene dose. There is an increased frequency of the allelic IGHG2*n gene in patients with increased IgE > 600 kU/l and IgE > 1000 kU/l, 76% and 88%, respectively, but also in patients with increased IgG4 > 1 g/l, 94% compared to 46% in healthy ones. The homozygous IGHG*bfn/*bfn diplotype with excluded IGHG3*g, IGHG1*a and IGHG2*-n genes and total loss of IgG3*g, IgG1*a and IgG2*-n antibodies are found in the atopic phenotype. The *IGHG* genes are involved in the IgE sensitization process. Childhood asthma patients with increased IgE have increased IgG2*n levels. The influence of excluded IgG genes and loss of IgG antibodies on IgE mediated allergy has not been fully ruled out [12, 15]. More than 90% homozygous IGHG*bfn/*bfn diplotypes are found in hyper IgG4 syndrome.

### Excluded IGHG1*a Genes

#### In Diabetes Mellitus Type 1

In 36 children with diabetes type1, 59% expressed homozygous IGHG1*f/*f, 37% heterozygous IGHG1*f/*a and 6% homozygous IGHG1*a/*a. In the homozygous IGHG1*f/*f genotype, the IgG1*a molecules are excluded. A virus etiology has been proposed in diabetes type 1. IVIG treatment early in disease might be tried. Transplantation of B cells producing IgG1*a molecules might be tried-.

### Immunotherapy of Patients with Excluded IGHG(Fcγ)(GM) Genes

#### In Active Immunotherapy. Vaccination

Homozygous IGHG2*-n/*-n genes with excluded IGHG2*n genes are low responders to polysachaaride antigens in pneumococcal vaccination while the alternative homozygous IGHG2*n/*n genes with excluded IGHG2*-n are high responders [11]. The response of viral and bacterial vaccines of the 10 different individual *IGHG*(Fcý)(GM) diplotypes are probably variable and must be investigated further. The side effects of vaccines may be associated to the *IGHG*(Fcý)(GM) genes.

### In Passive Immunotherapy

#### By IVIG Treatment the Excluded IgG Subclass Genes-molecules are Supplied to Patients with Excluded IGHG(Fcγ)(GM) Genes

Commercial IVIG preparations contain all Fcγ variants. In IVIG treatment of patients with excluded IgG subclass genes, the missing IgG subclasses are supplied (Fig. 6). This finding is an evidence of the absolute differences of the 2 alternative GM alleles of the IgG subclasses.

4 patients with excluded IgG genes and loss of allelic IgG subclasses were treated with IVIG (Fig. 6). One patient with Common variable immunodeficiency (CVID) and one with hyper IgM (HIGM), both with excluded IGHG1*a and IGHG2*n genes were supplied with IgG1*a and IgG2*n molecules. One patient with IgG3 deficiency (IgG3D) with the homozygous IGHG*ga-n/*ga-n diplotype, with excluded IGHG1*f and IGHG2*n genes and was supplied with the IgG1*f and IgG2*n molecules. Another CVID patient with the IGHG*bf-n/*ga-n with excluded IGHG2*n genes, was supplied with IgG2*n molecules. The supply of GM-specific IgG subclasses were identified and trough levels assessed, mean of 8 determinations, during 8 months. The trough levels of given allelic IgG2*n molecules were increased compared to IgG1*a and IgG1*f molecules which indicate a prolonged survival time for the IgG2*n subclass in treatment with IVIG [6]. The identification of the supplied alternative allotypes is the proof of alternative innate IgG subclasses as different unique entities.

## Discussion

With a novel competitive ELISA technique it is possible to further dissect the GM genetic system, to further investigate IgG subclass immunity in health, disease and immunotherapy (Fig. 1). The IgG subclass genes are serologically assessed by GM allotypes, genetic markers of the Fcγ part, of the constant heavy G chains, *IGHG*(Fcγ)(GM) genes. With a novel competitive ELISA, the alternative GM allotypes, alleles of IgG3, IgG1 and IgG2, respectively, are identified, defining 6 precise IgG subclass genes, expressed as 6 innate IgG subclass molecules: IgG3*b &IgG3*g, IgG1*f & IgG1*a and IgG2*n & IgG2*-n [3] from chromosome 14q32.3. The alternative IgG subclass genes have different structures and function, are novel unique precise molecules, with different amino acids, different electrophoretic rates [5], different half-life-times [6], different developing rates [8], different frequencies in disease [9, 14], different responses by antigen stimulation of virus [10], bacteria [11] and allergens [12] and different impact on active and passive immunotherapy [20].

*IGHG*(Fcγ)(GM)genes are inherited according to Mendel with allelic exclusion and IgG3-IgG1 linkage disequilibrium. 4 different IgG3-IgG1-IgG2 haplotypes, 10 diplotypes and 10 innate lymphoid B cells are registered in the healthy population. With dissection of the IgG subclass genetic system it is possible to further investigate different IgG subclass immunity in disease and immunotherapy. In homozygous *IGHG*(Fcγ)(GM) IgG subclass genotypes the alternative IgG subclass genes are excluded with total loss of the alternative IgG subclass molecules, IEI. Total loss of IgG subclasses was disclosed and should not be mixed up with the “IgG subclass deficiency” defined as decreased serum IgG subclass levels.

In this report we focus on excluded IgG subclass genes. In homozygous IgG subclass genotypes (Table 1) the alternative IgG subclass genes are excluded with total loss of alternative IgG subclass molecules. The frequency of homozygous IgG subclass genes with excluded alternative IgG subclass genes and total loss of IgG subclass molecules are found significantly increased in severe viral and bacterial infections, primary immunodeficiencies, autoimmunity, allergy and malignancy. Excluded IgG subclass genes with loss of IgG subclass molecules, inborn errors of immunity are found in healthy (587 Caucasians):10.9% loss of IgG3*b & IgG1*f, 45.1% loss of IgG3*g & IgG1*a, 33.9% loss of IgG2*n and 20.1% loss of IgG2*-n (Table 1) possibly demonstrating the variable phenotypes in pandemics. Excluded IgG subclass genes deteriorate IgG immunity and are inborn errors of immunity sometimes in combination with monogenic PID diseases. Patients with excluded IgG subclass genes could be treated with IVIG, which contains all genetic variants, passive immunotherapy, with supply of missing antibodies (Fig. 6).

The alternative *IGHG2* gene variants are most interesting: IgG2*n and IgG2*-n molecules differ immunochemically and by one amino acid at CH2(282): the IGHG2*n gene contains Metionin and the alternative IGHG2*-n contains Valin. In vaccination with bacterial polysaccharide the homozygous IGHG2*n/*n genotypes with excluded IgG2*-n genes are found in the high specific antibody responding phenotypes and the opposite homozygous IGHG2*-n/*-n genotypes with excluded IgG2*n genes are found in the low specific antibody responding phenotypes. According to this, the innate immunity controls the adaptive immunity.

Homozygous IGHG2*-n/*-n, excluded IGHG2*n genes, with total loss of innate IgG2*n subclass molecules are found significantly increased in severe RSV infection, Kawasaki syndrome, various PIDs, autoimmunity and malignancy with IGHG2*-n gene dose dependency. IGHG2*-n is a risk gene and IGHG2*n is protective in viral disease.

Virus are active, as FcýRs with the Fcý part of innate IgG subclasses as targets. Virus discriminate innate IgG subclasses. Herpes simplex virus 1 (HSV1) is binding preferably to IgG1*a, Cytomegalovirus (CMV) preferably to IgG1*f and Hepatitis C virus (HCV) to both IgG1*f and IgG2*n molecules [22–24]. Gammaglobulin injections was prophylactic treatment for hepatitis A exposure, several years ago, before vaccination was introduced. Excluded IgG genes with total loss of innate IgG subclasses have impact on the susceptibility of viral disease and probably on prolonged viral disease. Recurrent HSV1 infections is common in homozygous IGHG1*f/*f genotypes with excluded IGHG1*a genes and loss of IgG1*a antibodies. Early IVIG treatment of one patient with IgG2 deficiency and loss of IGHG2*n genes with acute facial paresis, Bells paralysis, stopped the disease development. Such patients must be further investigated. IVIG treatment of PID patients prevented outbreak of Covid-19 [28].

The dominating homozygous IgG2*-n molecules with the total loss of IgG2*n molecules may be responsible for the RS viral infection. In Kawasaki syndrome the loss of IgG2*n molecules is successfully treated with IVIG. The early IVIG treatment with supply of IgG subclasses is successful in Kawasaki syndrome. IgG subclass genes control the survivals in Yellow fever epidemics [25] an effect in accordance with the Darwinian selection theory. The variable phenotypes of the population in pandemics may be explained by excluded IgG subclass genes. The same IgG subclass gene patterns are found in both severe viral disease and in PIDs and in autoimmunity which may explain post-infection symptoms, as post-Covid. Homozygosity of IGHG2*-n/*-n and excluded IgG2*n molecules in 34% of healthy, are risk genes to fall ill in severe viral infections but also severe bacterial infections, as septicaemia. It is suggested that excluded genes may cause lifelong survival of virus in humans. Assessment of *IGHG*(Fcγ)(GM) genes and identification of excluded genes can be used for diagnosis, prognosis and possible treatment with IVIG molecules as supply of the missing excluded IgG subclass antibodies. Excluded *IGHG*(Fcγ)(GM) genes are found in autoimmune diseases with possible initial viral infection but continuous morbidity because of inherited life-long inability to get rid of virus, as Herpes simplex virus attacks (mouth herpes) by excluded IGHG1*a genes. It is suggested that excluded GM specific IgG subclass genes cause lifelong survival of virus. Excluded IGHG2*n genes in 34% healthy, are risk genes to fall ill in severe viral pandemics, severe bacterial infections, as septicaemia and severe autoimmune disorders.

The IGHG*bf-n/*bf-n and IGHG*ga-n/*ga-n with homozygous IGHG2*-n/*-n and excluded IGHG2*n are significantly increased in low antibody responding genotypes, severe viral and bacterial infections, Kawasaki syndrome, various PIDs severe autoimmunity and malignancy. In contrast the IGHG*bfn/*bfn with homozygous IGHG2*n/*n and excluded IGHG2*-n are significantly increased in high antibody responding genotypes, IgE mediated allergy and hyper IgG4 syndrome. IGHG*bf-n/*bf-n and IGHG*ga-n/*ga-n both with homozygous IGHG2*-n/*-n are significantly increased in IgG2 deficiency and IgG3 deficiency, respectively.

The IGHG2*-n/*-n genes with excluded IGHG2*n are most important in both severe viral and bacterial infections and in low vaccine responding genotypes compared with the opposite excluded IGHG2*-n genes, in IgE mediated allergy and in high polysachaaride responding genotypes. The *IGHG*(Fcγ)(GM) genes have impact on active and passive immunotherapy. It was shown that diseases with excluded *IGHG*(Fcγ)(GM) genes and loss of IgG subclass molecules are treated with IVIG with supply of missing IgG molecules (Fig. 6). The evolutionary parental Mendelian inherited GM-IgG subclass genes and excluded GM- IgG subclass genes, IEI control human immunity.

The innate IgG subclasses play a critical role in orchestrating inflammation. IgG subclasses are crucial for various immune functions including neutralization, phagocytosis, antibody dependent cellular cytotoxicity and the complement system. IgG subclasses elicit distinct cytokine profiles by human myeloid human cells dependent on FcγR activation. Excluded IgG subclass genes with loss of innate IgG subclasses and loss of innate B cells deteriorate the human immune system. The hidden genetic GM markers of antibodies with im pact on both health and disease, can be disclosed by ELISA test to understand the IgG system better, representing a new cause of the inborn errors of immunity.

The alternative GM specific IgG subclass genes have different structures and functions and respond differently to antigen stimulation by virus, bacterias and allergens and active and passive immunotherapy.

Excluded IgG subclass genes with loss of IgG subclass molecules are also found in healthy (587 Caucasians): 10.9% loss of IgG3*b & IgG1*f, 45.1% loss of IgG3*g & IgG1*a, 33.9% loss of IgG2*n and 20.1% loss of IgG2*-n (Table 1) as inborn errors of immunity, with risk to get ill.

*IGHG2* genes are most interesting. Excluded IGHG2*n genes and total loss of IgG2*n molecules are found: in 55% of 49 patients RS virus infektions, in 55% of 49 patients with Kawasaki, in 59% of 233 patients with PIDs, in 76% of 33 patients with CVID, in 58% of 43 patients with IgG2D, in 57% of 54 patients with IgG3D, in 55% of 62 patients with IgAD, in 67% of patients with Wiscott Aldrich syndrome, in severe systemic JCA, and in non IgE mediated asthma. The opposite excluded IGHG2*-n genes, total of loss IgG2*-n and increased IgG2*n levels are found in IgE mediated asthma, in hyper IgG4 syndrome and in high responding phenotypes specific bacterial polysaccharide. Excluded IGHG1*a genes, total loss of IgG1*a molecules are found in 59% of 36 children with diabetes mellitus type1.

## Summary

A novel ELISA technique is used to assess GM allotypes of IgG subclass genes of B cells. The alternative genes are novel precise molecules IgG3*b & IgG3*g, IgG1*f & IgG1*a and IgG2*n & IgG2*-n, from chromosome 14q32.3, with Mendelian inheritance. The GM-specific IgG subclasses are unique molecules with different structures and functions, different responses by antigen stimulation of virus, bacteria and allergens and different impact on active and passive immunotherapy. In homozygous genotypes, the alternative GM specific GM allotypes are excluded a new example of inborn error of immunity (IEI). Homozygous genotypes with excluded GM allotypes are found in both healhy and disease, but excluded genes are significantly increased in diseases, in severe virus and bacterial infections, in autoimmunity, in PIDs and in malignancy. 4 IgG subclass haplotypes are also markers of B cells, 10 GM-specific IgG subclass genotypes are found in healthy individuals of the Caucasian population. Significantly increased numbers of patients are found with homozygous GM genotypes and excluded alternative IgG subclass genes, total loss of alternative GM-specific IgG subclass molecules, inborn errors of immunity, IEI. Significantly increased numbers of IgG2*-n/-n with excluded IgG2*n are found in RSV infection, in Kawasaki syndrome, in PIDs, in JCA, in septicemias and in chronic non atopic asthma. Increased homozygous are found IgG1*f/f with excluded IgG1*a in diabetes type 1. The opposite increased homozygous IgG2*n/n with excluded IgG2*-n found in IgE mediated asthma and hyper IgG4. The findings suggest a causal link between excluded GM-specific IgG subclass genes and immunological diseases, but associated to infectious agents, IVIG contain all GM-specific IgG subclass genes. IVIG therapy as a method, to supply excluded genetic GM variants, provides a new approach for clinical treatment and future IgG subclass studies. Transplantation of GM-specific B cells producing excluded GM genes might be a possibility.

## Data Availability

No datasets were generated or analysed during the current study.
